# Long axial field of view (LAFOV) PET-CT: implementation in static and dynamic oncological studies

**DOI:** 10.1007/s00259-023-06222-3

**Published:** 2023-04-20

**Authors:** Antonia Dimitrakopoulou-Strauss, Leyun Pan, Christos Sachpekidis

**Affiliations:** Clinical Cooperation Unit Nuclear Medicine, German Cancer Research Center, Im Neuenheimer Feld 280, D-69120 Heidelberg, Germany

**Keywords:** Imaging, LAFOV PET-CT scanners, Oncology

## Abstract

Long axial field of view (LAFOV) PET-CT scanners have been recently developed and are already in clinical use in few centers worldwide. Although still limited, the hitherto acquired experience with these novel systems highlights an increased sensitivity as their main advantage, which results in an increased lesion detectability. This attribute, alternatively, allows a reduction in PET acquisition time and/or administered radiotracer dose, while it renders delayed scanning of satisfying diagnostic accuracy possible. Another potential advantage of the new generation scanners is CT-less approaches for attenuation correction with the impact of marked reduction of radiation exposure, which may in turn lead to greater acceptance of longitudinal PET studies in the oncological setting. Further, the possibility for the first time of whole-body dynamic imaging, improved compartment modeling, and whole-body parametric imaging represent unique characteristics of the LAFOV PET-CT scanners. On the other hand, the advent of the novel LAFOV scanners is linked to specific challenges, such as the high purchase price and issues related to logistics and their optimal operation in a nuclear medicine department. Moreover, with regard to its research applications in oncology, the full potential of the new scanners can only be reached if different radiopharmaceuticals, both short and long-lived ones, as well as novel tracers, are available for use, which would, in turn, require the appropriate infrastructure in the area of radiochemistry. Although the novel LAFOV scanners are not yet widely used, this development represents an important step in the evolution of molecular imaging. This review presents the advantages and challenges of LAFOV PET-CT imaging for oncological applications with respect to static and dynamic acquisition protocols as well as to new tracers, while it provides an overview of the literature in the field.

## Introduction

Positron emission tomography (PET) was introduced in the late 1980s for oncological studies, mainly employing the radiotracer 2-deoxy-2-[fluorine-18] fluoro-D-glucose ([^18^F]FDG) [1]. The first generation of PET scanners for clinical use had a very limited axial field of view (FOV) of about only 3 cm, dictated by the complexity and overall cost of the system. Since then, several technological developments took place over the years involving a gradual increase of the FOV up to approximately 25 cm, depending on the vendor, and the introduction of new faster scintillators, such as lutetium-oxyorthosilicate (LSO) or lutetium yttrium orthosilicate (LYSO) crystals. The next milestone in the evolution of PET systems was the development of the first commercial hybrid scanners allowing simultaneous morphologic/functional imaging, shorter attenuation correction, and exact anatomic localization of the PET findings. Indeed, at the turn of the twenty-first century, the first PET-CT scanners were introduced and were readily implemented into clinical routine for diagnosis, staging, and therapy response assessment in the oncological setting [2], while approximately 10 years later, simultaneous hybrid PET and magnetic resonance imaging (PET-MRI) was introduced for clinical use but has not been as widely applied in oncologic imaging as PET-CT [3]. The recent development of fully digital PET-CT systems with the replacement of photomultiplier tubes by silicon photomultipliers (SiPM) was a great step forward, overcoming many of the limitations encountered with previous generation scanners and leading, among others, to improved time of flight (TOF) performance and better methods for image reconstruction [4–7]. Nowadays, the newest generation of commercially available PET-CT scanners provides a long axial field of view (LAFOV), ranging approximately between 1 and 2 m, and offers several advantages as compared with the older scanners with a more limited FOV of about 15–25 cm, enabling, apart from the achievement of larger anatomical coverage, an increase in system sensitivity [8, 9].

The advent of LAFOV PET-CT scanners is associated with a variety of advantages mainly including a higher sensitivity and increased lesion detectability due to the larger FOV and the increase of counts and measured coincidences. High sensitivity whole-body (WB) imaging is important for oncological patients but also for inflammatory diseases. Another advantage is the reduction in acquisition time of static images and/or the reduction of the administered radiopharmaceutical dose leading to less radiation exposure for both patients and personnel. Several review articles have discussed the advantages of total-body PET-CT at a theoretical level and for different applications [10–12]. In the next sections, the preliminary results of the application of the LAFOV systems as well as some considerations linked to their application in clinical practice will be briefly discussed.

## Improved image quality due to higher sensitivity and increased lesion detectability

The high sensitivity of the new systems allows improved image quality and lesion quantification, which in turn lead to increased lesion detectability. Alberts et al. compared the performance of the LAFOV Biograph Vision Quadra PET/CT (10-min acquisitions) and the standard axial field-of-view (SAFOV; 2-min per bed position) Biograph Vision 600 PET/CT system in a group of 44 oncological patients with different tumors studied with three different radiotracers ([^18^F]-FDG, [^18^F]-PSMA-1007, [^68^Ga]-DOTATOC) applied at standardized doses (3.5 MBq/kg of [^18^F]-FDG, 250 MBq [^18^F]-PSMA-1007, 150 MBq [^68^Ga]-DOTATOC) [13]. In a head-to-head comparison under clinical conditions, they demonstrated an increased sensitivity of the LAFOV scanner as reflected by the higher signal-to-noise ratio (SNR) and target lesion-to-background ratio (TBR), and the improved image quality. Prenosil et al. compared the sensitivity of the aforementioned systems and reported that the sensitivity based on National Electrical Manufactures Association (NEMA) measurements was 5 times higher for the LAFOV system, when image reconstruction was based on a maximum ring difference (MRD) of 85 (corresponds to an acceptance angle of 18°) and about 10 times higher in MRD 322 (corresponds to an acceptance angle of 52°) and comparable to the uExplorer (FOV: 194 cm) due to the comparable acceptance angle for axial line of responses (LORs) [7].

## Reduction in acquisition time and/or administered dose

The increased sensitivity of LAFOV PET-CT scanners may alternatively allow for shorter acquisition times and/or low-dose examination protocols. Although it is still unclear what is the optimal acquisition time or radiotracer dose for a whole-body PET-CT examination with the new systems, several studies have highlighted the possibility of marked reduction of acquisition time and dose without a clinically relevant decrease in image quality. Hu et al. were the first to evaluate the feasibility of ultra-low tracer activity in a cohort of 30 oncological patients undergoing total-body PET/CT with the uEXPLORER scanner after injection of 0.37 MBq/kg [^18^F]-FDG [14]. PET raw data were acquired within 15 min and reconstructed using data from the first 1, 2, 4, 8, and 10 min and the entire 15 min. The authors were able to show no significant difference in TBR and liver SNR among all the images acquired for 8 min or longer. Moreover, a sub-cohort of 11 patients with colorectal cancer (CRC) was compared with a matched group of CRC patients who received a full [^18^F]-FDG activity (3.7 MBq/kg) with an acquisition time of 2 min. This matched-pair study revealed no significant difference in the image quality score and quantitative parameters between the ultra-low-activity group with an 8-min acquisition and the full-activity group with a 2-min acquisition. In the study by Alberts et al., the LAFOV Biograph Vision Quadra system could deliver images of comparable quality and lesion quantification in under 2 min, compared to routine SAFOV acquisition (16 min for equivalent FOV coverage) [13]. By analogy, if the LAFOV scans were maintained at 10 min, proportional reductions in applied radiopharmaceutical could obtain equivalent lesion integral activity for activities under 40 MBq quality.

Further, Tan et al. compared the performance of total-body PET/CT (uEXPLORER scanner) after application of half-dose [^18^F]-FDG activity (1.85 MBq/kg; 15-min), with conventional PET/CT (uM780 scanner; 2-min per bed position) and full-dose [^18^F]-FDG in a cohort of 56 lung cancer patients [15]. The 15-min acquisition time of the LAFOV group was split into 4-min and 2-min duration groups. Image quality scores and liver SNR in the 2-min acquisitions with the LAFOV system were significantly higher than those in the full-dose group examined with the conventional scanner. Importantly, all lesions detected with 15-min acquisitions could be also identified by 2-min and 4-min acquisitions. Using again low-dose (1.85 MBq/kg) [^18^F]-FDG activities and the uEXPLORER scanner, the same group explored the boundary of acquisition time for total-body PET/CT oncological imaging with a LAFOV system [16]. After 15-min total-body PET acquisitions, the initially acquired images were reconstructed and further split into 15-, 8-, 5-, 3-, 2-, and 1-min duration groups. They showed that acquisition times ≥ 5 min could provide comparable lesion detectability and sufficient information as regular protocols, showing, moreover, better compatibility and feasibility with clinical practice. Based on these findings, the authors proposed a 5–8 min PET protocol as an optimized acquisition time range using half-dose (1.85 MBq/kg) [^18^F]-FDG activity for the needs of oncological diagnosis.

In line with the previous, our group aimed to determine an appropriate acquisition time range for the LAFOV Biograph Vision Quadra PET/CT using low-dose (2 MBq/kg) [^18^F]-FDG activity in a group of 49 melanoma patients [17]. After the performance of 10-min PET acquisitions, the PET data were reconstructed and further split into 8-min, 6-min, 5-min, 4-min, and 2-min duration groups. Although as a general trend the reduction of acquisition time was associated with a decrease of liver SNR and TBR, we could demonstrate that a 5-min static acquisition of the torso provides comparable diagnostic quality to standard lengths of acquisition.

These preliminary results indicate that the shortening of PET acquisition times is not associated with a concomitant, clinically relevant decrease of diagnostic performance and may be very practical for busy departments, since it would allow an increase of the number of exams and higher patient throughput. At the same time, such shortened protocols can improve patient comfort and considerably prevent motion artifacts. Of course, the potential for a marked increase in the number of scanned patients would be inevitably associated with some required modifications related to logistics and infrastructure of a nuclear medicine department. These would include, among others, an increase in preparation, application and changing rooms for the patients, more personnel (both technical and medical) as well as a potent radiopharmaceutical department for the accompanying increased demand in tracer doses.

Furthermore, the reduction of the applied radiopharmaceutical doses represents a dosimetric advantage for both patients and personnel. Less radiation exposure is important with respect to longitudinal PET-CT studies within therapy response assessment and in particular for studies early in the course of treatment. Longitudinal PET studies will very likely gain acceptance and find their role both within routine therapy monitoring and also within clinical trials, given the fact that PET studies are more sensitive in evaluating short-term therapeutic effects compared to morphological imaging modalities [18]. Treatment stratification based on PET-CT is an important field in oncological management with promising results and significant therapeutic and prognostic implications in the entire spectrum of patient management, as recently highlighted in the field of immunotherapy [19, 20].

## Delayed scanning—low-dose imaging

Late images have been proposed for distinction between malignant and inflammatory lesions and for yielding a better TBR depending on the tumor type and the radiotracer applied. In a recent paper on [^68^Ga]-PSMA-11 PET-CT in recurrent prostate cancer, the employment of a LAFOV PET-CT system rendered 4 h late imaging feasible and demonstrated, as comparable to standard 1 h images, an improved TBR and improved SNR with a borderline significance at 4 h imaging while visual inspection was only modestly impaired [21].

In this context, an important application of the new scanners would be late imaging after the application of PET radionuclides with a longer half-life for imaging of slower biological processes, like antibodies, which show slower clearance and not a rapid uptake. Such examples are [^89^Zr]-labeled radiopharmaceuticals, which are coming up. [^89^Zr] has a physical half-life of 3.3 days and [^89^Zr]-labeled antibodies represent highly promising agents for cancer immunotherapy monitoring. Some of these agents are already in a limited clinical use and include [^89^Zr]-atezolizumab, [^89^Zr]-anti-CD8 minibody as well as [^89^Zr]-trastuzumab [22–25].

## CT-less approaches for attenuation correction

**Fig. 1:**
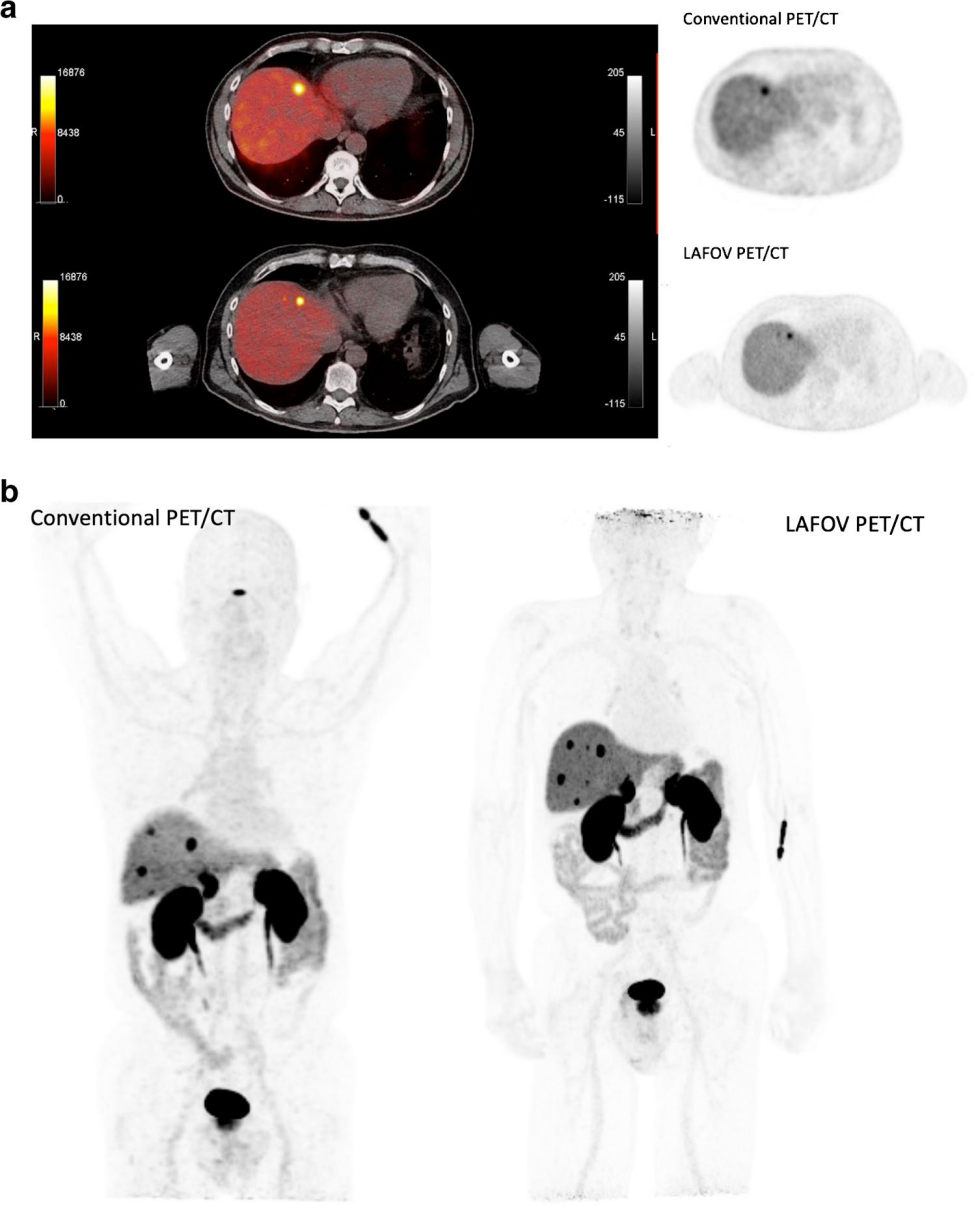
**a** Comparison of a [^68^Ga]-DOTATOC study in a patient with liver metastases of a neuroendocrine carcinoma with a conventional PET-CT scanner 1 h p.i. (Biograph mCT, upper row) and a LAFOV PET-CT system 2 h p.i. (Biograph Vision Quadra, lower row). Apart from the larger metastatic lesion in the cranial part of the organ, a second smaller liver metastasis is delineated with the LAFOV scanner in the transversal slices despite the delayed scanning due to the higher sensitivity of the new system. **b** Comparison of the maximum intensity projection (MIP) images of the same patient with both scanners

**Fig. 2:**
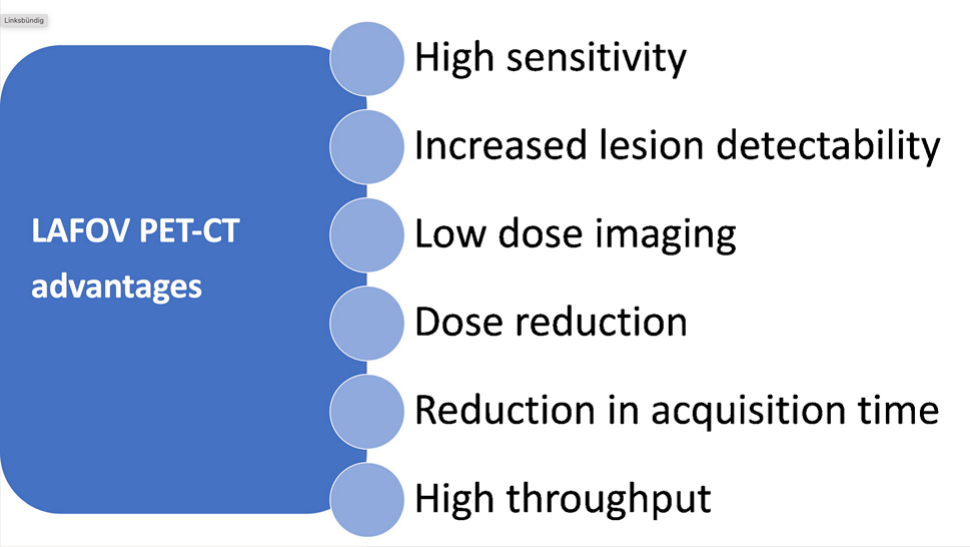
Summary of the advantages of LAFOV PET-CT scanners

Recent works have reported the feasibility of CT-less approaches for PET attenuation correction using the LSO intrinsic radiation of LAFOV PET-CT scanners to create an initial estimate of the attenuation maps. In particular, Teimoorisichani et al. created improved attenuation maps based on joint activity and attenuation reconstruction algorithms and compared to CT-based PET images [26]. The comparison of both approaches demonstrated a 6.5–8.3% average quantitative error. The same group applied this approach in 18 oncological patients and demonstrated that the mean absolute errors in SUV between the CT-based reconstructed images and the CT-less images were less than 5% in healthy organs, less than 7% in brain gray matter, and 4.3% for all evaluated tumors [27]. Another promising AI-based approach for CT-less attenuation correction for LAFOV PET-CT scanners has been published by Xue et al. [28, 29]. Such approaches may be interesting for longitudinal PET studies within very short time intervals for serial therapy response assessment (Fig. 1a, b and Fig. 2).

## Optimization of dynamic PET imaging

### Large FOV

**Fig. 3:**
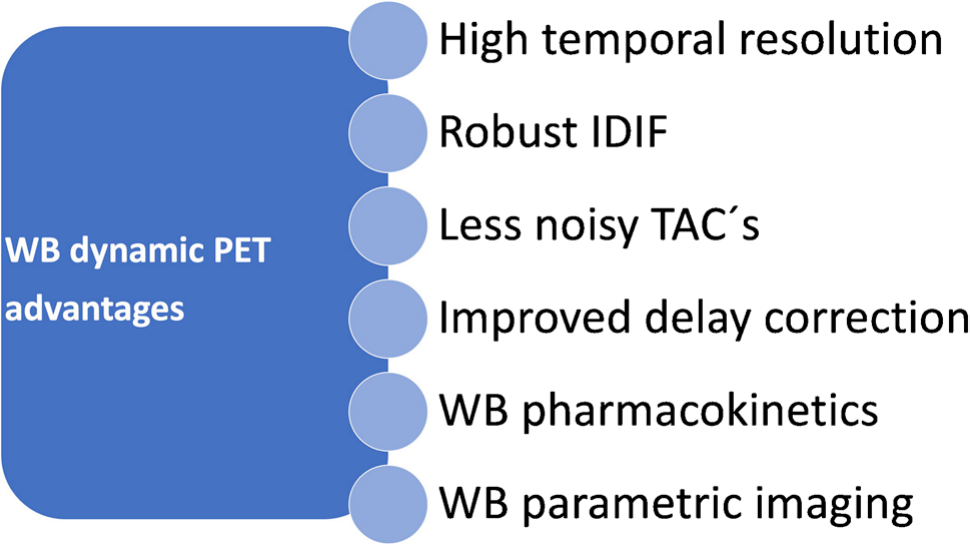
Summary of the advantages of LAFOV PET scanners in terms of whole-body (WB) dynamic PET imaging

Dynamic PET imaging (dPET) is still mainly an experimental tool for oncological applications hampered until now by some technical limitations of conventional scanners, such as the limited FOV, the limited time resolution in particular of short images, as well as the lack of operator-friendly and robust evaluation software programs for image segmentation and tools for the evaluation of the dynamic series in a reproducible way. However, dPET may prove helpful when applied additionally to conventional, static WB imaging in several settings, like tumor diagnosis, staging, and therapy response assessment [30]. The LAFOV PET-CT scanners have several advantages, which may facilitate the use of dPET imaging. The main advantages of LAFOV PET-CT specifically in terms of dPET imaging are summarized in Fig. 3.

**Fig. 4:**
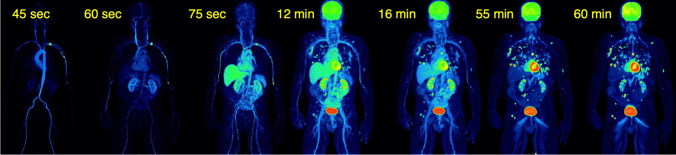
Dynamic [18F]-FDG PET-CT study in a patient with metastatic melanoma. Serial torso images from 45 s to 60 min p.i. demonstrate the high temporal resolution of the LAFOV scanner in the first seconds of dPET acquisition with the delineation of the large arterial vessels as well the gradual increase of the tracer uptake in the tumor lesions up to 60 min. PET angiography based on early dynamic PET-CT images may become an option for vascular evaluation, given the satisfying spatial and temporal resolution of the LAFOV systems

With the LAFOV PET-CT scanners, it is not necessary to define a FOV for the dynamic acquisition since the body trunk can be assessed with only one measurement. This means that an evaluation of radiotracer kinetics of all organs can be performed simultaneously. Especially in patients with extended metastatic disease, it is for the first time possible to measure simultaneously the tracer uptake in practically all tumor lesions and all organs (Fig. 4). This aspect is important particularly for therapy monitoring, drug development, and investigation of possible interactions between different organs, known as connectomes. Connectomes seem to have an impact also for non-oncologic applications. In a recent review paper, it was postulated that Parkinson disease (PD) is highly heterogenous and consists of several subtypes, which can be divided into a peripheral nervous system (PNS)-first and a central nervous system (CNS)-first subtype [31]. In this context, WB PET-CT studies may be helpful to identify the subtype of PD.

### Improved temporal resolution

LAFOV scanners provide a higher time resolution than conventional systems, which means that it is easily possible after the list mode acquisition to reconstruct very short images defined by the users with a good image quality. Exemplary, at out institution we use a Biograph Vision Quadra (Siemens Healthineers) and reconstruct normally 33 frames with an increasing frame duration, starting from the time of tracer injection up to 60 min post-injection (p.i.) for [^18^F]- and [^68^Ga]-labeled radiopharmaceuticals (10 × 15 s, 5 × 30 s, 5 × 60 s, 5 × 120 s, 8 × 300 s).

### Improved quality of image-derived-input-function

Manual blood sampling at different time points p.i. as well as continuous automatic blood sampling during the whole dynamic acquisition is considered as the gold standard for input function calculation [32]. However, this method has several limitations: it is invasive, it is associated with potential complications (arteriospasm, hematomas, nerve damage etc.) and sampling errors, and it increases patient discomfort. Therefore, in clinical practice, arterial sampling is not routinely used within an imaging study. With the advent of the new LAFOV scanners, the quality of the image-derived-input-function (IDIF) required for pharmacokinetic modeling is much more robust due to the fact that the heart and all large vessels like the aorta are within the FOV for dPET. The LAVOF PET-CT scanners will facilitate IDIF calculations by placing a volume-of-interest (VOI) in a large vessel, like the descending aorta. This was a major problem with conventional scanners, in particular when the dPET study was performed in areas without a large vessel in the FOV, like brain studies or studies of the lower abdomen, the pelvic area, and the extremities. Attempts to overcome the limitations of the use of IDIF of smaller vessels have been made, like partial volume correction or use of population-based input function, which is a normalized average of measured arterial blood samples from several subjects [33, 34]. Time-activity-curves (TACs) of noisy data from small vessels have been fitted by using a sum of up to three decaying exponentials to reduce noise [35, 36]. Furthermore, identification and correction of IDIF delay in different target and reference VOIs is an important aspect of pharmacokinetic modeling, leading to more robust results. IDIF delay influences the calculation of kinetic data meaning that the use of TAC’s without delay correction may be wrong. This effect was studied by Wang et al. for total-body multiparametric imaging of tumors for parametric compartment modeling. The authors concluded that the time delay varies in different organs and lesions and that consideration of time delay for IDIF improved multiparametric imaging [37].

Automatic image segmentation of the whole vessel is possible with new segmentation algorithms. This will further improve the whole evaluation procedure by yielding more robust input data. Automatic segmentation algorithms will improve the VOI-based image analysis not only of the input function but also of target and reference VOIs, and will lead to more robust and standardized image analysis and data evaluation. Attempts are undertaken to provide AI-based image segmentation based on both PET and CT data to provide an automatic segmentation of functional tumor volume, which is important in particular for longitudinal studies. Segmentation techniques include PET-based threshold methods as well as radiomic features from CT. Machine learning and deep learning models based on neural networks are utilized for the classification of the images and for segmentation of suspicious regions of interest [38, 39]. The idea is to develop decision support systems based on AI for a better classification of suspicious PET findings.

### Possibility of shortened acquisition protocols

Another aspect is the use of shortened acquisition protocols. The use of dPET will be facilitated by the LAFOV PET-CT scanners due to the better characteristics of the scanner, like the enhanced sensitivity and temporal resolution, needed for the early frames of a dPET acquisition. However, new shorter protocols are needed to facilitate its clinical use. One approach may be a short acquisition (0–15 min) starting with the tracer injection followed by a late acquisition 50–60 min p.i. depending on the tracer used [40]. Moreover, recently, the feasibility of performing accurate [^18^F]-FDG Patlak analysis using scaled population-based input functions with only 20 min of PET data from a LAFOV PET scanner was demonstrated [41].

### Applications in compartment modeling

Compartment modeling has its roots in pharmacology and biochemistry and has the goal to estimate biologically relevant parameters, which provide information about the pharmacokinetics of a radiotracer. It has been for several years used in PET for the characterization of tracer kinetics. The basic principles of kinetic modeling and parametric imaging, its clinical applications, and its limitations are described in detail elsewhere [42, 30].

The introduction of LAFOV PET-CT scanners has allowed for the first time the performance of whole-body pharmacokinetic studies, better temporal resolution, more robust IDIF calculation, and less noise in the TACs of the target and reference VOIs. This is particularly important for the assessment of new radiopharmaceuticals and evaluation of possible interactions between different organs. However, many known limitations of the compartment modeling approach still exist. Such a limitation is the fact that the assessment of the transport rates is operator-dependent due to the use of an iterative fitting to calculate the least squares between measured and model data, which may lead to overfitting problems and lack of reproducibility. The use of machine learning approaches will be still needed for robust and reproducible results that are user-independent for both VOI-based and pixel-wise quantitative analysis of PET data. Details about this method and an overview about the different algorithms which are in use for compartment modeling are described elsewhere [43, 44].

### Applications in parametric imaging

**Fig. 5:**
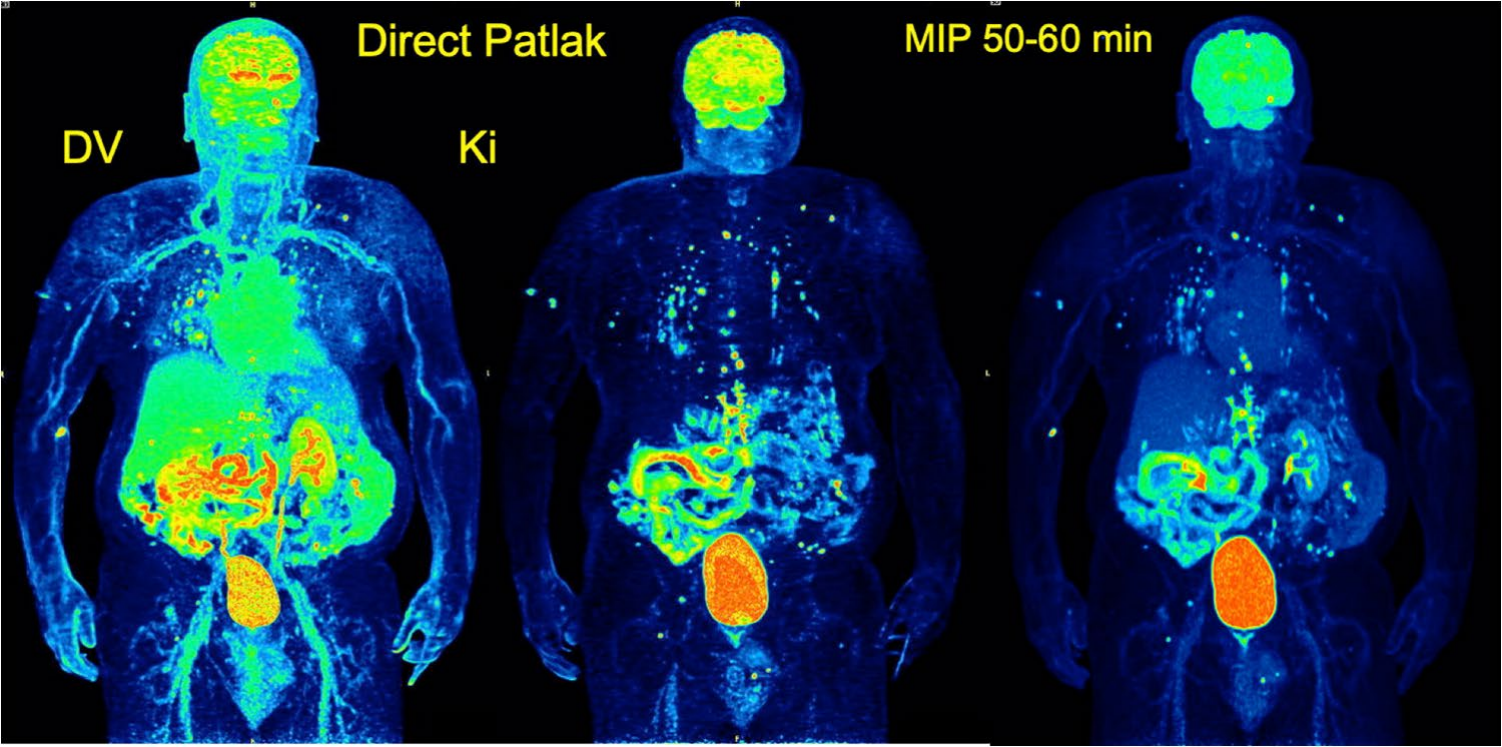
Direct Patlak images of [^18^F]-FDG PET in a patient with metastatic melanoma. At the right side, the maximum intensity projection (MIP) image after reconstruction of the PET data acquired at 50–60 min p.i. demonstrates multiple hypermetabolic metastatic lesions. At the left side, the corresponding distribution volume (DV) and influx (K_i_) parametric Patlak images are depicted

Parametric imaging is a method of feature extraction allowing the visualization of an isolated parameter of a tracer’s kinetics based on dedicated mathematical models and a voxel-wise calculation, instead of a VOI-based analysis. The advantage in comparison to pharmacokinetic analysis is the direct visualization of different kinetic parameters, like tracer influx or transport rates (K_1_, k_2_ etc.), instead of providing their absolute numbers. The first step involves the selection of the appropriate model for parametric imaging depending on the tracer used. An overview of different models is provided elsewhere [42]. In general, there are models based on compartmental and noncompartmental approaches. Concerning the compartmental models, mostly postprocessing approaches have been used until now with the conventional PET-CT scanners, which use the reconstructed PET images for further analysis, e.g., for the calculation of Patlak images [45]. New software tools provided for the LAFOV PET-CT scanners allow the so-called direct image reconstruction of Patlak images, which means that the Patlak algorithm is implemented into the reconstruction software [46]. The reconstruction of parametric Patlak images additionally to static images has been used within research purposes mostly with [^18^F]-FDG. Two sets of images are calculated by this approach, the distribution volume (DV) images, which reflect the perfusion-related part of [^18^F]-FDG and the influx or K_i_ images, which reflect the phosphorylated part of the tracer [47]. The impact of the additional use of parametric Patlak images is still open and an issue of current research (Fig. 5). Dias et al. investigated the largest patient collective with 109 oncological tumor patients with a SAFOV digital PET system of 26 cm FOV by using a multibed protocol [48]. They could not find any significant differences in the number of pathological lesions detected by direct Patlak as compared to conventional static images; however, they reported a higher TBR and contrast-to-noise (CNR) ratio for K_i_ images as compared to SUV and four fewer false positive findings. Recently, Wang et al. presented parametric imaging results with the uExplorer total-body PET-CT scanner in a small cohort (5 cancer patients and 5 healthy volunteers) and concluded that modeling of the time delay of the blood input function and model selection is improved as compared to conventional PET-CT scanners [37].

Parametric Patlak images by deep learning methods without an input function have been reported recently by Li et al. [49]. The authors reconstructed direct Patlak K_i_ images from five-frame sinograms (25 min and 40–60 min post-injection), which served as an input, and reconstructed these images by a nested expectation maximization (EM) algorithm. They found a high correlation between the traditionally reconstructed Patlak images and the predicted K_i_ images based on their approach. However, these preliminary results need to be validated in prospective studies.

## New approaches for therapy assessment

The existing response criteria in radiology and nuclear medicine are mainly based on changes in the size and/or metabolism of target lesions (or other functional information depending on the applied tracer) as well as on the identification of new lesions in follow-up studies. Non-target lesions play a rather minor role. However, for more reliable response assessments of systemic cancer treatments, especially in the longitudinal setting, the accurate and reproducible calculation of whole tumor burden is necessary, rather than merely assessing target lesions. Some recently developed AI-based approaches for image segmentation will probably allow automatic, robust, and reproducible WB tumor volume calculations that may improve, standardize, and fasten therapy assessment and may lead to definition of new response criteria based on dedicated software programs. Together with the information on specific aspects of WB tumor pathophysiology, as reflected by radiotracer pharmacokinetic analysis, rendered for the first time feasible with dynamic LAFOV PET scanning, these approaches may potentially tailor therapy monitoring aiding in the direction of personalized oncological management.

## Conclusion—future aspects

The novel LAFOV PET-CT scanners represent an important step in the evolution of PET imaging, offering many advantages as compared to conventional systems. Regarding their clinical application in oncology, the main strength of the new systems is the higher sensitivity, leading to increased lesion detectability, faster scanning times, and lower administered tracer doses as highlighted in several studies. Moreover, the potential of CT-less approaches for attenuation correction, resulting in a marked reduction of radiation exposure, as well as the possibility for the first time of whole-body dynamic imaging represent unique characteristics of the LAFOV PET-CT scanners. On the other hand, this development is not without its challenges, including, among others, a high purchase price and issues related to logistics, personnel, and infrastructure of a nuclear medicine department. Finally, regarding its research applications in oncology, the full potential of the new scanners can only be reached if different radiopharmaceuticals, both short and long-lived ones, as well as novel tracers, such as new peptides and minibodies, which provide information about specific molecular tumor characteristics, are available. This would, of course, also require the appropriate infrastructure in the area of radiochemistry, such as on-site cyclotrons and more good manufacturing practice (GMP) radiopharmaceutical laboratories.

## Data Availability

Not applicable.
